# Association of daily physical activity level with health-related factors by gender and age-specific differences among Korean adults based on the sixth (2014-2015) Korea national health and nutrition examination survey

**DOI:** 10.20463/jenb.2017.0026

**Published:** 2017-06-30

**Authors:** Nana Chung, Hun-Young Park, Mi-Young Park, Yoon-Young Hwang, Chi-Ho Lee, Jin-Soo Han, Jaemoo So, Jisu Kim, Jonghoon Park, Kiwon Lim

**Affiliations:** 1. Physical Activity and Performance Institute (PAPI), Konkuk University, Seoul Republic of Korea; 2. Department of Physical Education, Konkuk University, Seoul Republic of Korea; 3. Department of Physical Education, Korea University, Seoul Republic of Korea

**Keywords:** Physical activity level, Lifestyle diseases, Health-related factors, Gender and agespecific difference

## Abstract

**[Purpose]:**

This study examined the effects of daily physical activity level on health-related factors according to gender and identified age-specific differences among Korean adults.

**[Methods]:**

Using data from the Korea National Health and Nutrition Examination Survey VI (2014-2015), we selected adults aged 19-64 years who participated in both a health examination and health interview survey. The study included 6,457 participants 19-64 years of age (2,611 men, 3,846 women).

**[Results]:**

Assessment of the differences in health-related factors according to age and physical activity in men and women by repeated two-way analysis of variance (ANOVA) revealed significant interaction effects on total cholesterol (TC) and triglyceride (TG) levels and diastolic blood pressure (DBP) in male participants, but there were no significant interaction effects for any health-related factors in female participants. The group of female participants aged 40-64 years with daily physical activity levels over 200 kcal showed a significantly increased prevalence of 46% for dyslipidemia compared to that in female participants with daily physical activity levels below 200 kcal. Physical activity was positively correlated with weight and high-density lipoprotein cholesterol (HDL-C) levels in men 19-39 years of age, compared to weight, waist circumference (WC), body mass index (BMI), and DBP in men 40-64 years of age, and weight, WC, BMI, glycated hemoglobin (HbA1c) and triglyceride (TG) levels in women 19-39 years of age. In women 40- 64 years of age, physical activity was especially significantly positively correlated with weight, BMI, HDL-C and negatively correlated with fasting glucose and TG levels.

**[Conclusion]:**

In male and female participants, the 40-64-year age group showed negative results for health-related factors compared to those in the 19-39-year age group. The higher the weight, WC, BMI, the higher is the physical activity level. Physical activity levels were significantly positively correlated with health-related variables.

## INTRODUCTION

Given their enormous health and economic burdens, particular attention needs to be paid to the current prevalence of lifestyle diseases and its components. Physical inactivity is one of the leading risk factors for global mortality and is increasing in many countries, adding to the burden of lifestyle diseases also known metabolic syndrome (MetS)^1^. MetS is characterized by a cluster of metabolic risk factors, including abdominal obesity, insulin resistance or glucose intolerance, atherogenic dyslipidemia, and hypertension. Individuals with MetS are at an increased risk of coronary heart and other diseases^2-5^. The body responds to physical activity in ways that have important positive effects on the musculoskeletal as well as the cardiovascular, respiratory, and endocrine systems^6^. These changes are consistent with a number of health benefits, including reduced risks of premature mortality, coronary heart disease, hypertension, and diabetes^7-11^. Thus, there is strong evidence that regular and adequate levels of physical activity at all ages reduces body mass index (BMI), body fat mass, low-density lipoprotein cholesterol (LDL-C), blood glucose, and blood pressure and habitual physical activity has been associated with improvements in many health outcomes^7,12,13^. Although a consensus is growing on the importance of the relationship between physical activity and health, the specific level of physical activity necessary for health benefit remains unclear^14^. There is continued debate regarding how much, what type, how often, what intensity, and the duration of physical activity. Nevertheless, according to the recommends of the American College of Sports Medicine (ACSM)^15^ the Surgeon General’s Report^12^, and the Centers for Disease Control and Prevention (CDC)^16^, adults can derive general health benefits from at least 30 minutes of moderate-intensity activity most days of the week. These levels of activity translate to burning around 150-200 calories per day^17^. 

In Korea, there are also efforts to prevent MetS. As part of these efforts, the Korea National Health and Nutrition Examination Survey (KNHANES) is an ongoing surveillance system that assesses the health and nutritional status of Koreans, monitors trends in health risk factors and the prevalence of major chronic diseases, and provides data for the development and evaluation of health policies and programs in Korea. The KNHANES has sufficient numbers of participants and age diversity to effectively examine age and gender differences in physical activity level. In addition, the medical examinations performed as part of the KNHANES included measurement of data on body weight, waist circumference (WC), and BMI as criteria for obesity; fasting glucose and glycosylated hemoglobin (HbA1c) levels as criteria for diabetes mellitus; total cholesterol (TC), high-density lipoprotein cholesterol (HDL-C), LDL-C, and triglyceride (TG) levels as criteria for dyslipidemia; and systemic blood pressure (SBP) and diastolic blood pressure (DBP) as a criteria for hypertension, all of which were also required to define MetS. 

However, most studies based on the KNHANES have focused on the relationship between MetS and the type, intensity, or frequency of physical activity per week^18-22^. To our knowledge, no study has evaluated the differences in physical activity according to age and gender level based on daily calorie consumption (kcal per day) in a population-based sample of Korean adults. Therefore, the purpose of this study was to evaluate the effects of daily physical activity level on health-related factors among Korean adults. To address the study aims, the effects of daily physical activity level on health-related factors were analyzed in gender- stratified young (19-39 years) and middle-aged (40- 64 years) groups because the incidence of MetS increases after 40 years of age. 

## METHODS

### Participants

We performed a secondary analysis of data from the 2014-2015 KNHANES. In brief, the KNHANES is a nationwide cross-sectional survey to assess the health and nutritional status of the Korean population. The Korea Centers for Disease Control and Prevention (KCDC) conducted the KNHANES. Participants were recruited using stratified, multistage cluster sampling. The current study used raw data from the KNHANES VI without subject contact. After obtaining permission from the KCDC, data were directly downloaded and password protected. The clinical and biochemical characteristics of the study populations are shown in Table 1. A total of 6,457 study participants 19-64 years of age (2,611 men, 3,846 women) were included. Their mean age was 39.98 years (44.01 and 44.28 years for men and women, respectively). There were many statistically significant differences in the clinical characteristics of health-related factors and daily physical activity (kcal) between men and women due to the anatomical and physiological differences between the sexes, starting with basic morphological differences. 

**Table 1:** Participant characteristics.

Variables	Males (n = 2,611)	Females (n = 3,846)	p-value
Age	44.01 ± 13.02	44.28 ± 12.39	0.396
Height	171.54 ± 6.43	158.45 ± 5.79	<0.001
Weight	72.29 ± 11.61	58.21 ± 9.10	<0.001
WC	85.61 ± 8.96	77.64 ± 9.28	<0.001
BMI	24.51 ± 3.36	23.19 ± 3.53	<0.001
Fasting glucose	101.66 ± 24.09	95.97 ± 20.48	<0.001
HbA1c	5.74 ± 0.94	5.62 ± 0.79	<0.001
TC	189.73 ± 34.85	189.51 ± 34.38	0.806
HDL-C	47.82 ± 11.24	56.01 ± 12.92	<0.001
LDL-C	115.05 ± 32.17	114.69 ± 31.02	0.735
TG	168.52 ± 138.40	108.95 ± 76.39	<0.001
SBP	118.77 ± 14.07	112.28 ± 15.58	<0.001
DBP	78.56 ± 9.92	73.15 ± 9.66	<0.001
Physical activity	309.67 ± 489.59	187.34 ± 294.21	<0.001

Data are M ± SD, WC = waist circumstance, BMI = body mass index, HbA1c = glycated hemoglobin, TC = total cholesterol, HDL-C = high-density lipoprotein cholesterol, LDL-C = low-density lipoprotein cholesterol, TG = triglyceride, SBP = systolic blood pressure, DBP = diastolic blood pressure.

### Main outcome measures

Estimates of lifestyle disease risk factors were performed. Age and gender-stratified models were used to further explore whether physical activity level was associated with lifestyle disease risk factors including body weight; WC; BMI; fasting glucose, glycosylated hemoglobin (HbA1c), TC, HDL-C, LDL-C, and TG levels; SBP; and DBP. 

### Calculation of daily physical activity levels. 

To examine age and gender-related differences in the physical activity variables, participants in the survey sample were categorized into four groups: male participants 19-39 and 40-64 years of age and female participants 19-39 and 40-64 years of age. To measure physical activity in adults, the WHO has developed the Global Physical Activity Questionnaire (GPAQ)^17^. This questionnaire was developed and validated by the WHO to systematically monitor global physical activity levels as one of the main lifestyle disease risk factors^23^. It is included in the Physical Activity through Sustainable Transport Approaches (PASTA) online questionnaire in order to collect information on the duration and frequency of physical activity during work, transportation, and leisure time. Screening of GPAQ data and calculation of physical activity as metabolic equivalent (MET) minutes per week, minutes per day, and sedentary minutes per day was performed according to the GPAQ analysis guidelines^24^. MET is the ratio of the work metabolic rate to the resting metabolic rate. One MET is defined as the amount of oxygen consumed while sitting at rest and is equal to 3.5 mL O_2_ per kg body weight x min. The estimated physical activity is expressed in MET units, as a product of the following parameters: the MET factor, the number of days performed per week, and the duration in minutes per day. One MET is also equivalent to a caloric consumption of 1 kcal/kg/hour. The respondents were divided into one of two activity categories: under or over 200 kcal per day. 

#### Equation:

1 MET = 3.5 mL O_2_/kg/min. 

L/min to kcal/min = multiply LO_2_/min x 5. 

## MET-min/week → Kcal/day. 

Calculation of daily physical activity level = {## x (3.5 x body weight) x 0.005 } ÷ 7. 

### Statistical analysis

All statistical analyses were conducted using IBM SPSS Statistics for Windows, version 23.0 (IBM Corp., Armonk, USA). Data are presented as means ± SD. Kolmogorov-Smirnov tests were used to ensure that all data had a normal distribution. After classifying all participants into two age groups and two levels of physical activity, the prevalence of various lifestyle disease risk factors according to physical activity level per day was measured. For this purpose, two-factor analysis of variance (ANOVA) (age × physical activity) was used to examine the interaction and main effect on various lifestyle disease risk factors. In addition, chi-square (X^2^) tests were used to investigate the prevalence of various lifestyle disease risk factors in men and women according to two levels of physical activity per day and the odds ratios were calculated. Finally, correlation analysis was performed using Pearson’s correlation coefficient between physical activity and health-related variables for each gender and age. A priori, the level of significance was set at 0.05. 

## RESULTS

### Differences in health-related variables according to age and physical activity level in men. 

Table 2 shows the differences in health-related variables according to physical activity level and age in male participants. The repeated two-way ANOVA analysis revealed significant interaction effect (p <0.038) in TC, TG, and DBP. Among male participants 19-39 years of age, daily physical activity level over 200 kcal was associated with more effective improvement in TC, TG, and DBP. The other health-related factors did not show significant interaction effects between physical activity level and age, but significant main effects between physical activity (p <0.039) and weight, BMI, fasting glucose level, and HbA1c level were observed, as well as between age (p <0.004) and weight; WC; levels of fasting glucose, HbA1c, TC, HDL-C, and TG; SBP; and DBP. Daily physical activity level over 200 kcal was associated with higher weight and BMI and lower fasting glucose and HbA1c levels. The 40-64-year age group showed lower weight, but presented negative results in most health-related factors compared to those of 19-39-year age group. 

**Table 2:** Differences in health-related variables according to physical activity and age in men.

Variables	Age	Daily physical activity		p-value	
		<200 kcal/day	200 kcal/day<		
Weight	19-39	74.77 ± 15.28	76.82 ± 12.57	Physical activity Age Interaction	<0.001 <0.001 0.370
	40-64	70.69 ± 10.04	73.88 ± 10.43		
WC	19-39	85.88 ± 11.51	85.53 ± 9.36	Physical activity Age Interaction	0.466 <0.001 0.146
	40-64	87.07 ± 7.82	88.12 ± 7.83		
BMI	19-39	24.70 ± 4.53	24.93 ± 3.58	Physical activity Age Interaction	0.008 0.481 0.166
	40-64	24.57 ± 2.96	25.31 ± 2.95		
Fasting glucose	19-39	96.54 ± 21.85	93.26 ± 14.11	Physical activity Age Interaction	0.039 <0.001 0.734
	40-64	108.93 ± 29.65	106.57 ± 24.96		
HbA1c	19-39	5.45 ± 0.83	5.37 ± 0.63	Physical activity Age Interaction	0.044 <0.001 0.655
	40-64	5.99 ± 1.12	5.86 ± 0.95		
TC	19-39	190.38 ± 35.28	185.03 ± 33.91	Physical activity Age Interaction	0.490 <0.001 0.038
	40-64	194.57 ± 36.28	197.24 ± 36.55		
HDL-C	19-39	46.37 ± 10.90	48.01 ± 10.35	Physical activity Age Interaction	0.255 0.004 0.104
	40-64	45.64 ± 11.44	45.35 ± 10.37		
LDL-C	19-39	115.13 ± 29.32	112.47 ± 30.52	Physical activity Age Interaction	0.899 0.105 0.157
	40-64	115.48 ± 33.22	117.70 ± 32.10		
TG	19-39	193.88 ± 177.24	158.73 ± 125.64	Physical activity Age Interaction	0.059 <0.001 0.024
	40-64	207.95 ± 158.52	211.06 ± 154.17		
SBP	19-39	115.92 ± 11.56	115.47 ± 11.05	Physical activity Age Interaction	0.981 <0.001 0.561
	40-64	121.29 ± 14.86	121.70 ± 14.75		
DBP	19-39	77.84 ± 10.04	75.72 ± 8.84	Physical activity Age Interaction	0.425 <0.001 0.001
	40-64	79.86 ± 9.58	81.16 ± 9.11		

Data are M ± SD, WC = waist circumstance, BMI = body mass index, HbA1c = glycated hemoglobin, TC = total cholesterol, HDL-C = high-density lipoprotein cholesterol, LDL-C = low-density lipoprotein cholesterol, TG = triglyceride, SBP = systolic blood pressure, DBP = diastolic blood pressure.

### Differences in health-related variables according to age and physical activity level in women. 

As shown Table 3, there was no significant interaction effect for any health-related factor. However, significant main effects between physical activity (p <0.042) were observed in weight, WC, BMI, and age (p <0.002) in all dependent variables. Daily physical activity level over 200 kcal was associated with higher weight, WC, and BMI and the 40-64-year age group showed negative results for all health-related factors compared to those of the 19-39-year age group. 

**Table 3:** Differences in health-related variables according to age and physical activity in women.

Variables	Age	Daily physical activity		p-value	
		<200 kcal/day	200 kcal/day<		
Weight	19-39	57.03 ± 10.52	60.05 ± 10.09	Physical activity Age Interaction	<0.001 0.002 0.798
	40-64	58.72 ± 8.49	61.48 ± 9.11		
WC	19-39	74.96 ± 9.57	76.24 ± 9.20	Physical activity Age Interaction	0.042 <0.001 0.577
	40-64	80.32 ± 8.82	81.05 ± 8.84		
BMI	19-39	21.99 ± 3.71	222.75 ± 3.62	Physical activity Age Interaction	<0.001 <0.001 0.635
	40-64	23.90 ± 3.39	24.48 ± 3.42		
Fasting glucose	19-39	90.26 ± 13.82	90.66 ± 11.48	Physical activity Age Interaction	0.370 <0.001 0.203
	40-64	99.89 ± 22.09	97.60 ± 20.78		
HbA1c	19-39	5.27 ± 0.58	5.32 ± 0.57	Physical activity Age Interaction	0.708 <0.001 0.076
	40-64	5.77 ± 0.87	5.68 ± 0.75		
TC	19-39	183.07 ± 32.52	179.82 ± 30.53	Physical activity Age Interaction	0.211 <0.001 0.629
	40-64	199.225 ± 36.02	197.81 ± 33.89		
HDL-C	19-39	58.20 ± 13.02	56.83 ± 13.33	Physical activity Age Interaction	0.871 <0.001 0.080
	40-64	52.43 ± 13.03	53.57 ± 13.57		
LDL-C	19-39	106.21 ± 28.43	104.29 ± 27.63	Physical activity Age Interaction	0.180 <0.001 0.859
	40-64	120.09 ± 31.91	117.58 ± 28.99		
TG	19-39	97.63 ± 77.07	102.41 ± 116.03	Physical activity Age Interaction	0.899 <0.001 0.411
40-64	137.80 ± 92.64	134.30 ± 96.23			
SBP	19-39	104.87 ± 9.93	104.17 ± 8.86	Physical activity Age Interaction	0.264 <0.001 0.792
	40-64	118.15 ± 17.36	117.02 ± 16.30		
DBP	19-39	69.70 ± 8.46	69.42 ± 7.61	Physical activity Age Interaction	0.954 <0.001 0.548
	40-64	75.97 ± 10.14	76.31 ± 9.36		

Data are M ± SD, WC = waist circumstance, BMI = body mass index, HbA1c = glycated hemoglobin, TC = total cholesterol, HDL-C = high-density lipoprotein cholesterol, LDL-C = low-density lipoprotein cholesterol, TG = triglyceride, SBP = systolic blood pressure, DBP = diastolic blood pressure.

### Prevalence and odds ratio (OR, 95% confidence interval [CI]) of lifestyle disease risk factors according to daily physical activity. 

Table 4 shows the prevalence of various lifestyle disease risk factors according to physical activity per day in four groups (19-39 and 40-64-year-old men and women, respectively). Chi-square tests revealed a significant 46% increase in the prevalence of dyslipidemia in 40-64-year-old women with daily physical activity levels over 200 kcal (OR: 1.46, 95% CI: 1.10-1.96) compared to that of women in the same age group with daily physical activity level below 200 kcal. There was no significant difference in the prevalence of lifestyle risk factors according to daily physical activity in the remaining three groups. 

**Table 4:** Prevalence and odds ratios of lifestyle disease risk factors according to daily physical activity.

Gender	Age	Disease type	Daily physical activity			
			<200 kcal/day		200 kcal/day<	
			Distribution	Odds ratio	Distribution	Odds ratio
Male	19-39	Abdominal obesity	110/483 (22.8%)	1	87/443 (19.6%)	0.83 (0.60-1.14)
		Obesity	181/483 (37.5%)	1	159/443 (35.9%)	0.93 (0.72-1.22)
		Diabetes	306/483 (63.4%)	1	268/443 (60.5%)	0.89 (0.68-1.16)
		Dyslipidemia	398/483 (82.4%)	1	384/443 (86.7%)	1.39 (0.97-1.99)
		Hypertension	102/483 (21.1%)	1	92/443 (20.8%)	0.98 (0.71-1.34)
Male	40-64	Abdominal obesity	227/981 (23.1%)	1	122/529 (23.1%)	1.00 (0.78-1.28)
		Obesity	336/981 (34.3%)	1	208/529 (39.3%)	1.24 (1.00-1.55)
		Diabetes	615/981 (62.7%)	1	331/529 (62.6%)	1.00 (0.87-1.14)
		Dyslipidemia	815/981 (83.1%)	1	457/529 (86.4%)	1.29 (0.96-1.74)
		Hypertension	206/981 (20.9%)	1	98/529 (18.5%)	0.86 (0.65-1.12)
Female	19-39	Abdominal obesity	191/912 (20.9%)	1	94/415 (22.7%)	1.11 (0.84-1.46)
		Obesity	339/912 (37.2%)	1	158/415 (38.1%)	1.04 (0.82-1.32)
		Diabetes	558/912 (61.2%)	1	272/415 (65.5%)	1.21 (0.95-1.54)
		Dyslipidemia	762/912 (83.6%)	1	348/415 (83.9%)	1.02 (0.75-1.40)
		Hypertension	175/912 (19.2%)	1	84/415 (20.2%)	1.07 (0.80-1.43)
Female	40-64	Abdominal obesity	374/1,769 (21.1%)	1	125/534 (23.4%)	1.14 (0.91-1.44)
		Obesity	645/1,769 (36.5%)	1	207/534 (38.8%)	1.10 (0.90-1.35)
		Diabetes	1133/1,769 (64.0%)	1	352/534 (65.9%)	1.09 (0.89-1.33)
		Dyslipidemia	1475/1,769 (83.4%)	1	470/534 (88.0%)*	1.46 (1.10-1.96)*
		Hypertension	396/1,769 (22.4%)	1	113/534 (21.2%)	0.93 (0.74-1.04)

*Significant difference from <200 kcal/day, p <0.05.

**Table 5:** Correlation between physical activity and health-related variables according to gender and age.

	Physical activity			
Gender	Male		Female	
Age	19-39	40-64	19-39	40-64
Weight	0.083*	0.150*	0.169*	0.072*
WC	0.020	0.083*	0.148*	0.013
BMI	0.048	0.129*	0.155*	0.043*
Fasting glucose	-0.056	0.011	0.047	-0.046*
HbA1c	-0.005	0.030	0.074*	-0.029
TC	-0.013	0.040	0.001	-0.022
HDL-C	0.071*	0.011	-0.052	0.052*
LDL-C	-0.034	0.005	0.003	-0.047
TG	-0.051	0.031	0.064*	-0.057*
SBP	0.030	0.025	0.021	-0.014
DBP	-0.030	0.062*	0.019	0.002

*Significant correlation, p <0.05.

WC = waist circumstance, BMI = body mass index, HbA1c = glycated hemoglobin, TC = total cholesterol, HDL-C = high-density lipoprotein cholesterol, LDL-C = low-density lipoprotein cholesterol, TG = triglyceride, SBP = systolic blood pressure, DBP = diastolic blood pressure.

### Correlation between physical activity and health-related variables according to gender and age. 

Pearson’s correlation coefficient analysis of physical activity and health-related variables in the four groups revealed that physical activity was positively correlated with weight and HDL-C level in 19-39-year-old men, compared to weight, WC, BMI, DBP in 40-64-year-old men and weight, WC, BMI, and HbA1c and TG levels in 19-39-year-old women. In particular, among women 40-64 years of age, physical activity was significantly positively correlated with weight, BMI, and HDL-C level and negatively correlated with fasting glucose and TG levels (Table.5). 

## DISCUSSION

MetS is primarily composed of abdominal obesity, diabetes, glucose intolerance, dyslipidemia, and high blood pressure. Reduced daily activity level results in increased risks of health-related factors, which are associated with MetS. Despite the growing prevalence of MetS in Korea, information is lacking on gender- and age-specific patterns of MetS among Korean adults^25^. Gender and age are thought to be an important factor affecting MetS and its outcomes^26,27^. Despite a number of studies that have demonstrated differences in metabolism and its components that are dependent on gender and age^25,27,28^, limited information about gender and age differences in the characteristics of MetS and its components is available regarding the Korean adult population. In addition, the relationship between daily activity level and health-related factors is not well reported. Physical inactivity is a major public health problem and compelling evidence suggests that it is a contributing factor in MetS. Although the important role of physical activity in the control of MetS has been demonstrated in various studies^29-32^, no studies have evaluated the effects of daily physical activity level on health-related factors in Korean adults. This study is an authoritative, evidence-based document providing a comprehensive evaluation of age and gender physical activity level differences and relationships between daily physical activity level and the indicators influencing health among adults in Korea. 

According to the World Health Organization (WHO), individuals who are insufficiently active have a 20% to 30% increased risk of death, adding to the burden of MetS compared to those who are sufficiently active^33^. There is strong evidence that low levels of physical activity are correlated to weight, BMI, waist-to-hip ratio, blood pressure, insulin levels, and total and low-density lipoprotein (LDL) cholesterol levels in men^34-37^. The main message of this study is that Koreans can substantially improve their health by increasing their daily physical activity levels. 

Our results showed that female participants were likely to engage in physical activity (187.34 ± 294.21 kcal per day) that meets the ACSM recommendation( burning around 150-200 calories per day for health benefits) compared to male participants with daily activity levels of 309.67 ± 489.59 kcal (Tab.1). These results are consistent with those reported in descriptive epidemiological studies of physical activity, which have consistently reported that male participants are more active than female participants^25,28,38,39^. 

The difference in health-related variables according to physical activity level and age in male participants showed significant interactions between physical activity level and age for TC, TG, and DBP (Table.2). Male participants aged 19-39 years with daily physical activity level over 200 kcal were associated with more favorable health markers, including TC and TG levels and DBP. The other health-related factors (weight, BMI, fasting glucose, HbA1c) showed significant main effects between physical activity and age for weight; WC; fasting glucose, HbA1c, TC, HDL-C, and TG levels; SBP; and DBP. Daily physical activity level over 200 kcal was associated with increased obesity-related factors such as weight and BMI but lower diabetes-related factors such as fasting glucose and HbA1c levels (Table.2). Although many studies have reported the association between the increasing prevalence of MetS and increased BMI, the clinical limitations of weight and BMI should be considered. Weight and BMI do not reveal anything about the body composition (how much fat, muscle, and bone mass) or provide any indication of the distribution of fat among individuals^40^. Therefore, overweight and high BMI do not necessarily directly reflect health risks. 

Among male participants 40-64 years of age, daily physical activity level over 200 kcal was associated with lower weight but presented negative results for most health-related factors compared to those of participants 19-39 years of age. However, there were no significant interaction effects in any health-related factors in women, but significant main effects between physical activities and weight, WC, and BMI and between ages and all dependent variables were observed. Similar to men, daily physical activity level over 200 kcal as associated with higher weight, WC, and BMI and the 40-64-years group showed negative results for all health-related factors compared to those of the 19-39-years group (Table.3). These results were consistent with those of previous studies that reported that increased abdominal obesity paralleled the age-related increase in MetS prevalence in both men and women; however, this observation was associated with high levels of TG and fasting glucose only in women^41^. 

In addition, women 40-64 years of age with physical activity levels over 200 kcal per day had a significant 46% increased risk in the prevalence of dyslipidemia compared to those with daily physical activity levels below 200 kcal. Contrary to most other reports^42-45^, dyslipidemia was not common in the inactive group compared to the active group. One possible explanation is that women with dyslipidemia may be more active for their health. There was no difference in the prevalence of the other lifestyle risk factors (abdominal obesity, obesity, diabetes, and hypertension) according to daily physical activity (Table.4). 

In general, physical activity was positively correlated with weight and HDL-C in 19-39-year-old men, compared to weight, WC, BMI, and DBP in 40-64-yearold men and weight, WC, BMI, HbA1c, and TG in 19-39-year-old women. Among the 40-64-year-old women, physical activity showed an especially significant positive correlation with weight, BMI, and HDL-C level and negative correlation with fasting glucose and TG levels (Table.5). 

With the recent development of national policy documents on physical activity promotion^23^, the issue of low-level physical activity is now a political priority in many countries. Because of its numerous health benefits, increasing physical activity in the majority of the population should be a central component of public preventive health policy. Therefore, the results of our study are helpful for the identification of the relationship between physical activity level and health-related factors, providing a basis for the promotion of national health care and risk management of MetS. 

## CONCLUSION

Male participants aged 19-39 years with daily physical activity levels over 200 kcal showed positive results in TC, TG, and DBP. The 40-64-year age group showed negative results in health-related factors compared to those in the 19-39-year age group in both men and women. Regardless of age and gender, the greater the weight, WC, and BMI, the higher was the physical activity level. Physical activity levels were significantly positively correlated with health-related variables. In conclusion, we confirmed that higher physical activity levels represent positive health-related factors and were effective for lifestyle disease in all participants. 

## LIMITATIONS

Data for the primary indicator were obtained subjectively using the GPAQ during the KNHANES. The GPAQ uses only self-reported occupational or leisure- time physical activity as the exposure variable. Because self-reports of complex and repetitive lifestyle behaviors are crude and imprecise, it is difficult to determine the exact type, amount, and intensity of physical activity that are associated with the observed health benefits. Indeed, recent evidence indicates that self-report methods may greatly underestimate group differences in physical activity levels. 
